# Nationwide epidemiological study of tuberculosis and other respiratory pathogens among children and adolescents in Brazil: TBPed Brazil study protocol

**DOI:** 10.1371/journal.pone.0342753

**Published:** 2026-02-12

**Authors:** Marcelo Comerlato Scotta, Márcia Polese-Bonatto, Fernanda Hammes Varela, Ivaine Tais Sauthier Sartor, Gabriela Oliveira Zavaglia, Caroline Nespolo de David, Ingrid Rodrigues Fernandes, Thais Raupp Azevedo, Annerose Barros, Anna Cristina C. Carvalho, Claudete Aparecida Araújo Cardoso, Clemax C. Sant’Anna, Andrea Maciel de Oliveira Rossoni, Marcelo Cordeiro-Santos, Afrânio Kritski, Fernanda Dockhorn da Costa, Nicole Menezes de Souza, Eduardo de Souza Alves, Heather J. Zar, Renato T. Stein

**Affiliations:** 1 Social Responsibility - PROADI-SUS, Hospital Moinhos de Vento, Porto Alegre, Brazil; 2 School of Medicine, Pontifícia Universidade Católica do Rio Grande do Sul, Porto Alegre, Brazil; 3 Infection Control Service, Hospital da Criança Conceição do Grupo Hospitalar Conceição, Porto Alegre, Brazil; 4 Programa de Pós-graduação em Pediatria e Saúde da Criança, Pontifícia Universidade Católica do Rio Grande do Sul, Porto Alegre, Brazil; 5 Laboratory of Innovation, Therapies, Teaching and Bioproducts, Oswaldo Cruz Institute, Fiocruz, Rio de Janeiro, Brazil; 6 Departamento Materno-Infantil, Faculdade de Medicina, Universidade Federal Fluminense, Rio de Janeiro, Brazil; 7 Faculdade de Medicina, Universidade Federal do Rio de Janeiro, Rio de Janeiro, Brazil; 8 Universidade Federal do Paraná, Curitiba, Brazil; 9 Gerência de Micobacteriologia, Fundação de Medicina Tropical Doutor Heitor Vieira Dourado, Manaus, Amazonas, Brazil; 10 Pós-Graduação em Medicina Tropical, Universidade do Estado do Amazonas (UEA), Manaus, Brazil; 11 Pós-Graduação em Saúde e Inovação, Universidade Nilton Lins, Manaus, Brazil; 12 Programa Acadêmico de Tuberculose, Faculdade de Medicina da Universidade Federal do Rio de Janeiro, Rio de Janeiro, Brazil; 13 General Coordination for Surveillance of Tuberculosis, Endemic Mycoses and Nontuberculosis Mycobacteria, Ministry of Health, Brasília, Brazil; 14 Department of Pediatrics and Child Health, Centre for Child & Adolescent Lung Health (CHILD), University of Cape Town, Cape Town, South Africa; Universiti Malaysia Sabah, MALAYSIA

## Abstract

The burden of lower respiratory tract infections (LRTIs) caused by *Mycobacterium tuberculosis* (MTB) among children and adolescents is often underestimated due to challenges in obtaining lower respiratory tract samples, nonspecific signs and symptoms, the paucibacillary nature of tuberculosis (TB), and the low yield of microbiological tests. Xpert MTB/RIF Ultra in induced sputum (IS) samples is the most promising test for improving microbiologic diagnosis of pulmonary tuberculosis (PTB) in young children. We describe the TBPed Brazil study, a multicenter, cross-sectional study in participants aged 6 months to 15 years. We designed a two-arm study: a hospital-based arm with patients hospitalized with LRTI and an outpatient-based arm with children and adolescents referred to TB-specialized clinics. The main aim in the hospital-based arm is to determine the prevalence of PTB. In the outpatient-based arm, the main objective is to determine the accuracy of Xpert MTB/RIF Ultra in IS samples compared to liquid culture. We evaluate in both arms: tuberculosis infection (TBI) prevalence, the accuracy of tongue swabs compared to liquid culture and clinical diagnosis, the risk factors associated with PTB or TBI; the accuracy of the Brazilian Ministry of Health scoring system for the diagnosis of PTB. In the hospital-based arm, we also evaluate the prevalence of viral and/or bacterial pathogens associated with LRTI. The sample size is 1,848 participants (1,118 hospitalized and 730 outpatients). Enrollment began in December 2021 and is expected to conclude in 2026, involving 27 study sites across Brazil. This is the first prospective nationwide investigation into the prevalence of PTB and TBI, as well as diagnostic accuracy across different methods, in children and adolescents in a large country with a significant TB burden. Study results will provide critical data on epidemiological, clinical, and diagnostic approaches to MTB and other respiratory pathogens in children in Brazil, guiding future studies and public health policies.

## Introduction

The burden of lower respiratory tract infections (LRTIs) due to *Mycobacterium tuberculosis* (MTB) among children and adolescents is usually underestimated, particularly in children under five years of age. In addition to the challenges of obtaining lower respiratory tract samples, nonspecific signs and symptoms, paucibacillary disease, and the low yield of microbiological testing contribute to its underdiagnosis and under-notification [1]. Most diagnoses of pulmonary tuberculosis (PTB) among children are presumptive, usually based on clinical and radiological prediction scores [2,3].

However, significant progress has been made in improving the microbiological diagnosis of PTB in children. The introduction of new molecular tests in clinical practice, such as Xpert МТB/RIF, has improved MTB detection in children [4]. Moreover, the development of a more sensitive Xpert MTB/RIF, i.e., the Ultra assay, which offers a 1–log improvement in the lower limit of detection, has increased diagnostic sensitivity across different clinical samples, such as nasopharyngeal, sputum, and stool [5–7]. Xpert MTB/RIF Ultra (Ultra) in induced sputum (IS) samples is the most promising for improving the diagnosis of PTB. In a large South African study, the sensitivity and specificity of the Ultra assay were 74.3% and 96.3%, respectively, when compared to liquid culture in a single IS sample. Two samples, IS and a nasopharyngeal aspirate, increased the sensitivity to 80% [8].

However, most studies originate from either low-income countries, particularly in Africa and Asia, or high-income countries [9–12]. In Brazil, one of the largest middle-income countries globally, tuberculosis (TB) has an overall intermediate incidence of 39.7 cases per 100,000 population in 2024 [13,14], which is high compared to high-income nations, but different from other lower and middle-income countries.

Current studies on PTB in Brazilian children and adolescents are based on retrospective data and do not evaluate the broader nationwide diversity [15,16]. The Brazilian Tuberculosis Pediatric (TBPed) study was planned on the assumption that PTB prevalence in children and young adolescents under 15 with LRTI would be between 2% and 3% and power calculations were based on these figures. Following the COVID-19 pandemic, new data suggest an increase in TB prevalence, due to poor treatment adherence (especially among adult patients), and higher rates of intra-domiciliary transmission [17–19].

The protocol includes children and young adolescents and was designed as a two-arm study in different settings. The first group consists of hospitalized participants with a diagnosis of LRTI, without necessarily a history or clinical signs and symptoms suggestive of TB. The second group consists of participants referred to TB-specialized clinics within the Brazilian public service TB network, to investigate and initiate treatment if the diagnosis is established.

The main aim of the hospital-based arm is to determine the prevalence of PTB among children and adolescents with LRTI. In the outpatient-based arm, the main objective is to evaluate Ultra’s accuracy in induced sputum samples compared to automated liquid culture. We assess in both study arms: tuberculosis infection (TBI) prevalence; the accuracy of Ultra in tongue swabs compared to sputum samples in Ultra and automated liquid culture; risk factors associated with PTB and TBI; accuracy of the Brazilian Ministry of Health scoring system for the diagnosis of PTB [20]. In the hospital-based arm, we also evaluate the prevalence of viral and/or bacterial pathogens associated with LRTI and describe the severity of hospitalized participants with LRTI without PTB.

## Methods

### Study design and setting

TBPed Brazil is a multicenter, cross-sectional study with prospective data collection. The study includes 27 sites in four of the five Brazilian regions (North, Northeast, Southeast, and South), in cities with a general incidence of TB higher than 50 cases per 100,000 population, or in TB clinics that serve as referral centers for TB patient care in the region. We excluded the Central-West region due to its lower TB incidence [21]. Enrollment began in December 2021 and is expected to be concluded in July 2026.

The study includes two different settings. In pediatric general hospitals, individuals hospitalized due to LRTI are eligible for enrollment. In outpatient referral TB clinics, individuals referred for PTB investigation are also eligible for enrollment.

## Eligibility

### Hospital-based study arm

#### Inclusion criteria.

Patients aged from 6 months to less than 15 years are eligible if admitted for less than 7 days, have a chest radiograph performed during the current admission, and present at least one of the following signs at enrollment:

- age-specific tachypnea (6 months to 1 year: 50 breaths per minute, 1–4 years: 40 breaths per minute; 5–14 years: 30 breaths per minute) or respiratory retractions and/or;
- lower respiratory signs of wheezing, crackling, or diminished breath sounds and/or;
- danger signs of severe pneumonia according to World Health Organization guidelines [22] and/or;
- radiographic signs suggestive of intrathoracic TB (tracheal compression and/or displacement; soft tissue opacity suggestive of adenomegaly; alveolar opacity; nodular infiltrate suggestive of miliary TB; cavitation; calcification of the lung parenchyma; or pleural effusion [23].

#### Exclusion criteria.

Participants are excluded if not living in Brazil, if previously treated or on treatment for any form of TB disease for at least 72 hours, have a previous diagnosis of cystic fibrosis or bronchiolitis obliterans, a tracheostomy, clinically diagnosed at enrollment with a healthcare-associated infection, asthma exacerbation without associated LRTI, or being on invasive or non-invasive mechanical ventilation. After inclusion, the participant is excluded if a sputum sample cannot be obtained.

### Outpatient-based study arm

#### Inclusion criteria.

Participants aged 6 months to 15 years at TB referral clinics are eligible if they have one of the following:

- persistent, unremitting cough for more than two weeks and/or;
- unexplained axillary temperature above 37.8 °C for more than one week and/or;
- persistent, unexplained lethargy and/or;
- decrease in playfulness/activity reported by the parent/caregiver and/or;
- weight loss/failure to thrive (weight loss is defined as >5% reduction in weight compared with the highest weight recorded in the last three months, and failure to thrive is defined as clear deviation from a previous growth trajectory), and/or documented crossing percentile lines in the preceding 3 months, and/or weight-for-age z score of ≤ −2 in the absence of information on previous/recent growth trajectory, and/or weight-for-height z score of ≤ −2 in the absence of information on previous/recent growth trajectory and not responding to nutritional rehabilitation (or antiretroviral therapy if HIV infected).

#### Exclusion criteria.

Participants are excluded if previously treated or on treatment for any form of TB disease for at least 72 hours, have a tracheostomy, or have a known impediment to collecting respiratory samples. After inclusion, the participant is excluded if a sputum sample cannot be obtained.

### Participant selection and study variables

**Fig 1:**
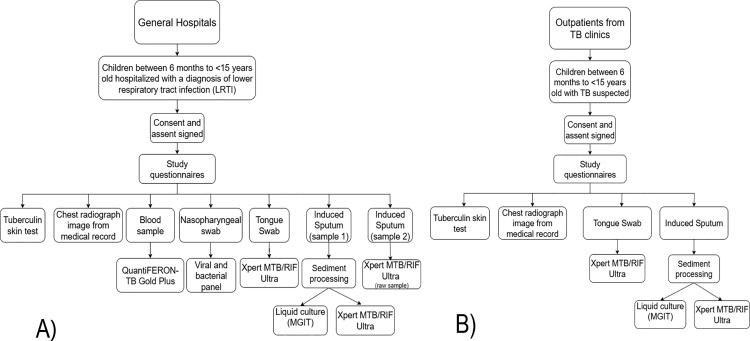
Study procedures performed in the hospital-based study arm (panel A) and in the outpatient-based study arm (panel B). MGIT = Mycobacteria Growth Indicator Tube.

On weekdays, a convenience sample is screened in both pediatric wards and outpatient clinics, based on the eligibility of potential participants. Potential participants’ legal caregivers are invited to be included. Study invitation and data collection is done by the research teams of the participating sites. To ensure consistency in all study procedures, the coordinating team trained all investigators and researchers involved in the study according to the Operational Manual, which contains the study’s standard operating procedures (SOPs). It required them to produce up-to-date Good Clinical Practice (GCP) certification. Study questionnaires are administered to the patient’s legal caregiver. Data collected in the TBPed Study are shown in Table 1. Fig 1 presents the study procedures performed in the hospital-based study arm (panel A) and in the outpatient-based study arm (panel B). Our study is cross-sectional; however, we conducted a small follow-up through medical record review to verify TB diagnoses and treatments administered after study entry. This medical record review is conducted for hospitalized participants at the time of hospital discharge or within 28 days of admission, whichever occurs first. Medical record review for outpatient participants occurs up to 60 days after study entry, the maximal time required for the release of liquid culture results.

**Table 1:** Data collected in the TBPed study.

**Socioeconomic and demographic data**
Child’s age; Skin Color/Race; Age of legal caregiver; Education level of legal caregiver; Number of people living in the same residence; Number of people sleeping in the same room as the child; Attends daycare or school; Anyone smokes in the house
**History of present illness**
Presence and time (days) of the following symptoms: cough (presence of phlegm, blood), shortness of breath, runny nose, loss of appetite, weight loss or failure to gain weight (<2 years), night sweats, fever, use of antibiotics to treat symptoms, improvement after antibiotic use
**Past medical history**
Birth weight, Gestational age at birth, Bronchiolitis in the first year of life, Hospitalization for bronchiolitis in the first year of life; Hospitalization for other respiratory problems; Disease compromising immunity; Chronic use of corticosteroids; Continuous medication use; Laboratory-confirmed COVID-19 diagnosis; Hospitalization for COVID-19; COVID-19 vaccination; HIV infection; Close contact with someone presenting cough; Close contact with someone diagnosed with TB; Previous preventive TB treatment; Up-to-date vaccination card
**Anthropometry and physical examination**
Weight, Height, Presence of BCG scar
**Collection of biological samples, chest radiograph, and TST**
Nasopharyngeal swab (hospitalized only); Lingual swab; Induced sputum, data about induced sputum safety; Blood (hospitalized only); Chest radiograph image collection; TST
**Medical record review**
TB diagnosis and treatment after study inclusion. Hospitalized only: Hospital admission date, ICU admission, HIV infection, use of ventilatory support, COVID-19 diagnosis upon admission, Use of antibiotics, Outcome of hospitalization (discharge, escape, death)

TB: tuberculosis, TST: tuberculin skin test, BCG: Bacillus Calmette-Guérin, ICU: intensive care unit, HIV: Human Immunodeficiency Virus, COVID-19: Coronavirus disease.

### Chest radiograph (CXR)

The CXR image is obtained from the participant’s medical record, as requested by the attending physicians. All researchers are trained in the Standardized Operational Procedure (SOP) for chest radiograph acquisition and storage in the study electronic case report form. The assessment of intrathoracic TB and pneumonia in children will be evaluated by consensus of two expert radiologists blinded to the clinical data, with a third reader available to resolve discordant decisions using a standardized reporting form. The criteria for radiological classification are described elsewhere [20,23–25].

### The Brazilian Ministry of Health’s score

**Fig 2:**
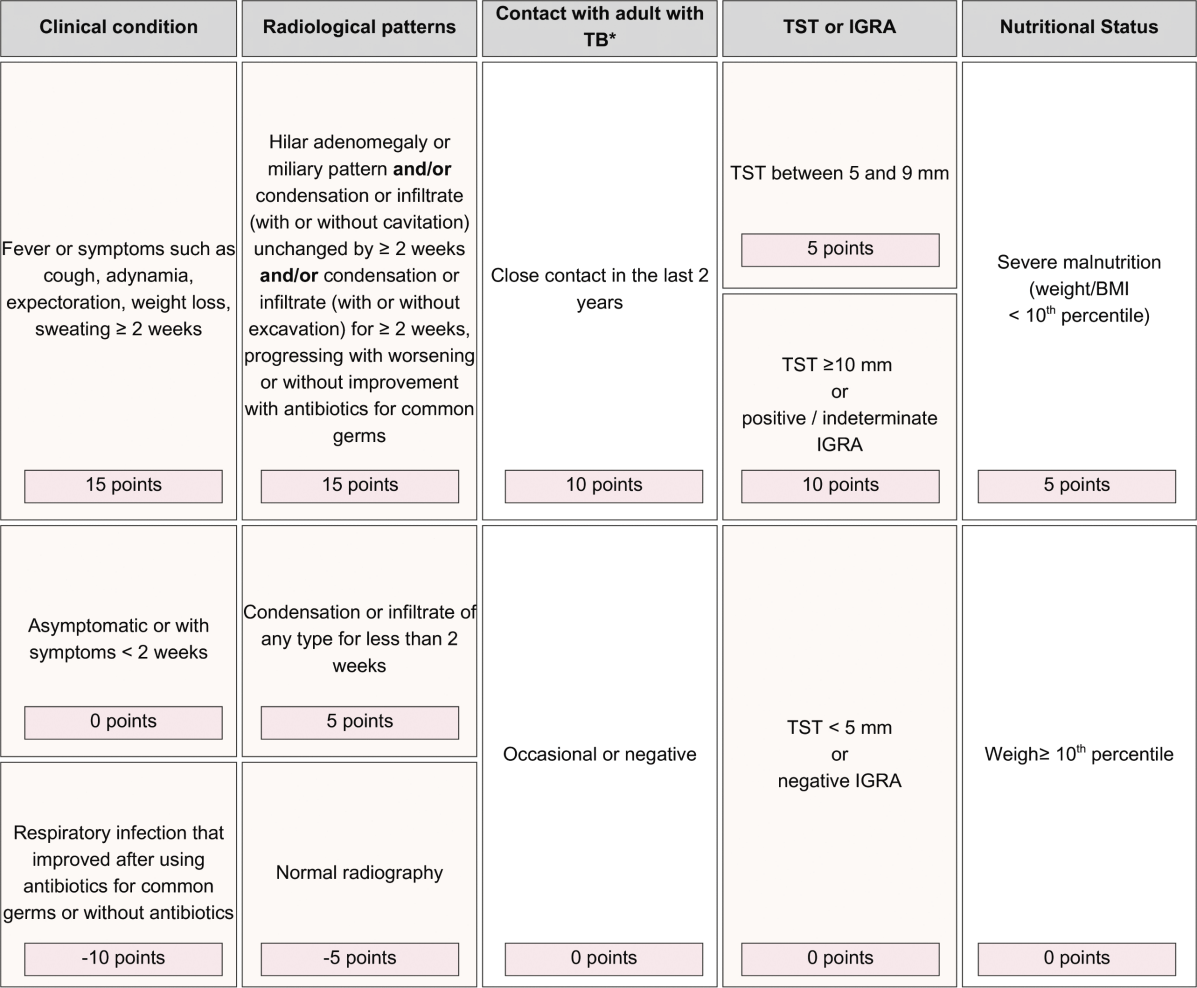
The Brazilian Ministry of Health’s score system for predicting PTB in children. RMT = rapid molecular test; TB = tuberculosis; TST = tuberculin skin test, IGRA = Interferon-Gamma Release Assay, BMI = Body Mass Index. More than or equal 40 points (very likely diagnosis) = it is recommended to start TB treatment; 30–35 points (possible diagnosis) = indicative of TB; it is advised to initiate treatment, at medical discretion; Less than or equal 25 points (diagnosis is unlikely) = investigation of the child should be continued.

The Brazilian Ministry of Health’s validated score [2,3] system for predicting PTB in children and adolescents, updated in 2022 [26], includes signs and symptoms, radiological findings, contact with adults with PTB, TST results, and nutritional status, as shown in Fig 2 and described elsewhere [3]. Diagnosis probability is classified as follows: at least 40 points = very likely diagnosis, 30–35 points = possible diagnosis, and 25 points or less = diagnosis is unlikely. The same score will be calculated using IGRA results instead of TST results when IGRA is available.

### TB-related diagnostic criteria

- Pulmonary tuberculosis (PTB):
  - Pulmonary tuberculosis confirmed: detection of MTB by liquid culture or by molecular biology tests;
  - Pulmonary tuberculosis - clinical diagnosis: TB clinical diagnosis by the attending physician without microbiological confirmation of MTB, adjudicated by the Brazilian Ministry of Health’s validated score system mentioned above, considering the diagnosis if at least 40 points;
- Tuberculosis infection (TBI): a TST (≥5 mm) or IGRA positive, and negative microbiological, radiological, and clinical diagnosis (excluding presumptive PTB or other forms of active TB).

### Study aims and outcomes

The main aim (primary outcome) in the hospital-based arm is to determine the prevalence of PTB. In the outpatient-based arm, the main aim (primary outcome) is to assess the accuracy of Ultra in IS samples compared to liquid culture. Table 2 describes the study aims and specifies the study arm involved, the statistical analysis plan, the missing data management, and the outcomes for each.

**Table 2:** Study aims, statistical analysis plan, missing data management, and outcomes.

Study aims	Study arm involved	Statistical analysis plan, missing data management, and outcomes
To evaluate the prevalence of PTB	hospital-based	Analysis will be conducted independently for each study arm. Eligible participants will be considered in the analysis. Analysis method: number of participants with PTB divided by the number of included ones. Elegibility criteria are described in the “Eligibility” section and PTB in the “TB-related diagnostic criteria” section. In the hospital-based arm, we will perform a sensitivity analysis using only the first sample results from Ultra to avoid imbalance in the prevalence estimates, as this population has a second sputum sample planned in the study protocol.
	outpatient-based	
To evaluate the prevalence of TBI	hospital-based	Analysis will be conducted independently for each study arm. Analysis method: number of participants with TBI divided by the number of included ones. In the hospital-based arm, participants with TST and/or IGRA results and without a PTB diagnosis will be included in the analysis. Participants without TST or IGRA results, or with indeterminate IGRA results, will be considered missing and excluded from analysis. In the outpatient-based arm, participants with TST results and without a PTB diagnosis will be included in the analysis. Those without TST results will be considered missing and excluded from analysis.
	outpatient-based	
To identify risk factors associated with PTB	hospital-based	Analysis will be conducted independently for each study arm. Demographic, socioeconomic, and household contact characteristics, time of symptom onset, and chest radiography results will be analyzed using logistic regression. Paired data will be used for analysis, and missing values will be excluded.
	outpatient-based	
To identify risk factors associated with TBI	hospital-based	Analysis will be conducted independently for each study arm. Demographic, socioeconomic, and household contact characteristics, time of symptom onset, and chest radiography results will be analyzed using logistic regression. In the hospital-based arm, participants without results from both TST and IGRA will be considered missing. Paired data will be used for analysis, and missing values will be excluded. In the outpatient-based arm, participants without TST results will be considered missing. Paired data will be used for analysis, and missing values will be excluded.
	outpatient-based	
To evaluate the accuracy of the Brazilian Ministry of Health score system for the prediction of PTB *versus* laboratory diagnosis (Ultra and/or liquid culture – obtained through sputum collection)	hospital-based	The analysis will be conducted jointly and independently for each study arm. Participants with at least one lab result (Ultra or liquid culture) and a score on the Brazilian Ministry of Health will be included in the analysis. Sensitivity, specificity, PPV, NPV, and 95% confidence intervals for all estimates will be calculated. Participants without a score on the Brazilian Ministry of Health and lab results (Ultra and/or liquid culture) will be considered missing. Paired data will be used for analysis, and missing values will be excluded.
	outpatient-based	
To compare the accuracy of the Brazilian Ministry of Health score system using either IGRA or TST *versus* laboratory diagnosis (Ultra and/or liquid culture – obtained through sputum collection)	hospital-based	Analysis will be conducted for the hospital-based study arm. Participants with results from TST or IGRA, and lab results (Ultra and/or liquid culture – gold standard) will be included in the analysis. Sensitivity, specificity, PPV, NPV, and 95% confidence intervals for all estimates will be calculated. Participants without TST or IGRA results, or lab results (Ultra and/or liquid culture), will be considered missing. Complete paired data are considered for analysis, and missingness will be excluded
To evaluate the accuracy of the IS of Ultra results *versus* the liquid culture results	hospital-based	The analysis will be conducted jointly and independently for each study arm. Participants with IS collection, Ultra results, and liquid culture results (gold standard) will be included in the analysis. Sensitivity, specificity, PPV, NPV, and 95% confidence intervals for all estimates will be calculated. Participants without lab results (Ultra and/or liquid culture) from the IS collection will be considered missing. Complete paired data are considered for analysis, and missingness will be excluded
	outpatient-based	
To evaluate the accuracy of the IS of Ultra results *versus* presumptive diagnosis or liquid culture results	hospital-based	The analysis will be conducted jointly and independently for each study arm. Participants with IS collection, Ultra results, and presumptive diagnosis plus liquid culture results (gold standard) will be included in the analysis. Sensitivity, specificity, PPV, NPV, and 95% confidence intervals for all estimates will be calculated. Participants without lab results (Ultra and/or liquid culture) from the IS collection will be considered missing. Paired data will be used for analysis, and missing values will be excluded.
	outpatient-based	
To evaluate the accuracy of Ultra results from tongue swab *versus* Ultra or liquid culture from sputum samples	hospital-based	The analysis will be conducted jointly and independently for each study arm. Participants with tongue swab results from Ultra and sputum results from Ultra or liquid culture (gold standard) will be included in the analysis. Sensitivity, specificity, PPV, NPV, and 95% confidence intervals for all estimates will be calculated. Participants without tongue swab results from Ultra or sputum results from Ultra or liquid culture will be considered missing. Paired data will be used for analysis, and missing values will be excluded.
	outpatient-based	
To evaluate the prevalence of detected viral and/or bacterial pathogens associated with LRTI	hospital-based	The analysis will be conducted for the hospital-based study arm. Participants with results from viral and/or bacterial panels will be included in the analysis. Analysis method: number of participants with pathogen infection divided by the number of included ones. Participants without results from the viral and/or bacterial panel will be considered missing and will be excluded.
To describe the severity of hospitalized participants with LRTI without PTB	hospital-based	The analysis will be conducted for the hospital-based study arm. Analysis method: number of participants with severity parameters divided by the number of included ones. Participants without information regarding: length of stay, use of supplemental oxygen, use of invasive mechanical ventilation, admission at ICU, and death will be considered missing, and will be excluded.

PTB: pulmonary tuberculosis, TBI: tuberculosis infection; TST: tuberculosis skin test, IGRA: Interferon-Gamma Release Assay, IS: induced sputum, LRTI: lower respiratory tract infection, PPV: positive predictive value, NPV: negative predictive value, ICU: intensive care unit.

### Specimen collection

Trained research health professionals undertake all the sample collection and laboratory procedures, according to the study’s SOPs. All samples are transported to partner laboratories by a specialized company, in accordance with biosafety standards.

### Tongue swab collection

Samples are collected using sterile swabs with an ejectable brush-shaped head convenient for DNA extraction (Whatman OmniSwab – Catalog number WB100035). The tongue swab sample is obtained before the sputum sample is collected. The collection is performed by brushing the swab on the tongue 6–8 times. After collection, the material is transferred to a 2 mL tube containing Tris-EDTA buffer (pH 8.0). The tongue sample is used for the Ultra test.

### Nasopharyngeal swab collection

The collection is performed with a flexible rayon swab, carefully introduced along the nasal septum until the nasopharynx is felt. It is rubbed, rotated, and held in the nasopharynx for a few seconds. The swab is removed, and the process is repeated in the other nostril. After collection, the swab is placed into Universal Transport Medium – Room Temperature (UTM-RT Copan) (Catalog number 330C) or saline solution (Catalog number SAL00013 bioBoaVista). After collection, the nasopharyngeal sample is stored at −80°C and analyzed to perform a viral and bacterial panel through qualitative reverse transcription-polymerase chain reaction (RT-PCR) assay.

### Induced sputum collection

All researchers involved in the study underwent standardized training in the induced sputum (IS) technique, following the study’s standard operating procedure, to ensure correct execution, participant safety, and site biosafety. As this technique is not widely used in Brazilian health services, such training was necessary to standardize collections and maintain sample quality, enabling researchers to collect specimens from all eligible participants in accordance with the study protocols.

Induced sputum training includes all steps of the technique, such as vital signs, pulse oximetry, and pulmonary auscultation. Then, two jets (100 µg) of Salbutamol with a spacer are administered before nebulization. Five mL of 5% hypertonic saline solution is nebulized for approximately 5 minutes. Nasopharyngeal aspiration (NPA) is performed to obtain secretions in all children aged 5 years or younger. Participants aged 5 years or older may have their samples collected through aspiration or spontaneously after induction. The study protocol does not require a minimum volume of sputum samples. Any volume obtained is sent for analysis. During the NPA procedure, the sputum could become trapped in the extension of the aspiration tube. To maximize sample use, the tube is washed at the end of the procedure by aspirating 3 mL of 0.9% saline solution into the collection bottle. As a standardization measure, the spontaneously expectorated samples are also diluted to 3 mL with 0.9% saline solution before being sent to the laboratory.

When possible, a second sample from hospitalized participants is obtained on the following day or at least 4 hours after the ﬁrst specimen. A clinical evaluation is performed at the end of the procedure, and the child is monitored for 30 minutes afterward. Possible adverse reactions, such as mild epistaxis, increased coughing, and wheezing responsive to an inhaled bronchodilator, are recorded during or after the procedure. The first induced sputum sample is used for the Xpert MTB/RIF Ultra and liquid culture. The second sample is used only for Ultra among hospitalized participants.

### Blood collection

Blood is collected in a 6 mL tube containing lithium or sodium heparin with a minimum volume of 4 mL. The tubes were packed in boxes at temperatures ranging from 17°C to 25°C for transfer to the processing laboratory, with a maximum storage time of 16 hours after collection. The blood samples are used for IGRA testing.

### Tuberculin skin test (TST)

A trained professional apply 0.1 mL of PPD-RT-23 (tuberculin purified protein derivative – manufacturer AJ vaccines A/S) subcutaneously to the arm and reads the skin test reaction. After administration, the reaction occurs between 48 hours and 72 hours (maximum 96 hours), and is measured in millimeters (mm) of induration (firm swelling). The test is considered positive if the induration is greater than or equal to 5 mm. Participants who previously tested positive are not tested again.

### Laboratory procedures (biological processing)

All sample processing and analysis procedures are conducted in a laboratory with trained technologists using standardized protocols.

### Sputum sediment processing

The sputum is stored in a cool box (2°C to 8°C) until it is transported to the laboratory, and the specimens are processed within at least five days of collection. Specimens were measured and decontaminated using either N-acetyl-L-cysteine and sodium hydroxide (final concentration of 1.0%) or BD BBL Mycoprep (Becton, Dickinson and Company, catalog number #240862), in a 1:2 volume ratio. The mixture was incubated at room temperature (20–30°C) for 15 minutes and then neutralized with sterile phosphate-buffered saline (PBS, pH 7.0) to a final volume of 50 mL. After centrifugation at 3000 × g for 15 minutes, the pellets are resuspended in 1.5 mL of PBS buffer and divided for testing with Ultra (Cepheid, Sunnyvale, CA) and automated liquid culture (BACTEC MGIT; Becton Dickinson, Franklin Lakes, NJ, USA).

### Sputum and tongue swab processing

The sputum and tongue swabs are maintained in a cold box (2° C to 8° C) until they are delivered to the laboratory, where the samples are handled within five days of collection. After homogenization, 1 mL of the sample is used for testing with Ultra (Cepheid, Sunnyvale, CA). This procedure is performed on the second sputum sample from hospitalized participants.

### Liquid culture - Mycobacteria growth indicator tube (MGIT)

A 0.5 mL volume of resuspended sputum pellet is inoculated into an automated liquid culture (BACTEC MGIT; Becton Dickinson, Franklin Lakes, NJ, USA). If positive, the corresponding cultured isolate will undergo phenotypic testing, according to the established routine by the laboratory, confirming the growth of MTB, phenotypic resistance testing is done by automated liquid MGIT culture, BD BACTEC™ MGIT™ 960 Sire kit (Becton, Dickinson and Company, catalog number #245123). Liquid cultures are considered negative after 42 days of incubation without isolation of any *Mycobacteria*.

### Molecular rapid test – Xpert MTB/RIF Ultra – sputum sediment

For sputum sedimented samples, 0.7 mL of the resuspended pellet (previously described in the sputum sediment processing) is added to 1.4 mL of the sample reagent included in the Ultra kit. Then, vortexed for 10 seconds, incubated at room temperature (20–30 °C) for 10 minutes, shaken again for 10 seconds, and incubated for 5 minutes (incubate for another 5 minutes if the sample is not completely liquefied). Two milliliters of the liquefied sample are slowly added to the Ultra cartridge and then inserted into the GeneXpert system to perform the test.

### Molecular rapid test – Xpert MTB/RIF Ultra – sputum and tongue swab

One milliliter of sputum or tongue swab is added to 2 mL of the sample reagent included in the Ultra kit. Following 10 seconds in the vortex, the sample is incubated at room temperature (20–30 °C) for 10 minutes, shaken again for 10 seconds, and incubated for 5 minutes (or for another 5 minutes if the sample is not completely liquefied). Two milliliters of the liquefied sample are slowly added to the Ultra cartridge and then inserted into the GeneXpert equipment to perform the test.

### Interferon-Gamma release assay – QuantiFERON-TB

Whole blood is collected as described in the blood collection. One milliliter (with a variation of 0.8 to 1.2 mL) is transferred to each QuantiFERON-TB Gold Plus tube kit (Nil, TB1, TB2, and Mitogen). To dissolve the antigens on the inner surface of the tube, homogenization is performed with a flat hand and uniform movements (exactly 10 inversions). The tubes are incubated at 37 °C in a vertical position for 16–24 hours and then centrifuged at 3000 × g for 15 minutes to separate the blood plasma. One hundred microliters of plasma are aliquoted for each tube and subsequently stored in a −80°C freezer until the QuantiFERON-TB Gold Plus ELISA is performed according to the manufacturer’s instructions.

Samples are analyzed in three research laboratories with expertise in the technique: the Immunology Laboratory at Fundação de Medicina Tropical Dr. Heitor Vieira Dourado (Manaus/AM), the Molecular Mycobacteriology Research Laboratory at Federal University of Rio de Janeiro Thoracic Diseases Institute (Rio de Janeiro/RJ), and the PROADI Research Laboratory at Moinhos de Vento Hospital (Porto Alegre/RS).

### Viral and bacterial panel

The RNA and DNA from nasopharyngeal swab samples are extracted using MagMax™ Viral/Pathogenic Nucleic Acid Isolation (catalog number A42352 Applied Biosystems) on the KingFisher Duo Prime System platform (ThermoFisher, USA). All samples are quantified using a NanoDrop™ Lite Spectrophotometer (ThermoFisher, Wilmington, Delaware, USA) and diluted to a concentration of between 1.0 and 2.0 ng/µL. The RT-PCR assay is done using Path™ 1-Step RT-qPCR Master Mix, CG (catalog number A15299, Applied Biosystems) and TaqMan™ Microbe Detection Assay (catalog number A50137) in 10 µL total reaction, with 5 µL of RNA. Five microliters (1 x 105 copies/μL) of TaqMan™ Respiratory Tract Microbiota Amplification Control (catalog number A39178) are used for reaction control.

The panel assess the presence of a range of common community-acquired respiratory pathogens: seven bacteria (*Bordetella pertussis, Chlamydophila pneumoniae, Haemophilus influenzae, Moraxella catarrhalis, Mycoplasma pneumoniae, Staphylococcus aureus and Streptococcus pneumoniae*) and 18 viruses (adenovirus 1 e 2; human bocavirus; human coronavirus types HKU1, 229E, NL63, and OC43; human enterovirus, influenza A virus types H1 and H3; influenza B virus; human metapneumovirus; human parainfluenza virus types 1, 2, and 3; human rhinovirus, respiratory syncytial virus (RSV) types A and B, and SARS-CoV-2 (*S*, *N* and *ORF1ab* genes). The samples are stored in different study sites and are transported to the PROADI Research Laboratory at Hospital Moinhos de Vento for analysis. Samples undergo quality control to verify transport time, temperature (if they remain frozen), tube identification, and volume, and are then stored at −80°C until analysis.

### Data collection and management

Standardized case report forms (CRFs) were developed to collect demographic, clinical, and laboratory data. Study’s CRFs are available in ReBEC storage (https://ensaiosclinicos.gov.br/welcome - U1111-1332–7450). Data collection is paper-based, based on interviews and medical reviews. After collection, all study data are entered, stored, and managed using an electronic database on REDCap (Research Electronic Data Capture) software, hosted on a secure server [27]. The data are maintained in a study database on REDCap, and the research team has access to the platform through registration and login.

### Blinding procedure

Radiologists are blinded to the clinical data and laboratory results during chest radiograph evaluation.

### Sample size

The sample size to assess the prevalence of PTB in children hospitalized due to LRTI was obtained based on a systematic review summarizing the prevalence of TB in children aged less than 5 years hospitalized due to LRTI. The average prevalence reported for laboratory-confirmed TB was 7.5% in settings with an overall yearly incidence usually higher than 90 cases per 100,000 population [28]. Moreover, in a study with the most similar inclusion criteria to this study, the prevalence of laboratory-confirmed TB in 2,439 participants was 7%, and for unconfirmed TB was 17% [29]. As the yearly incidence reported in this study setting (South Africa) was 406 cases per 100,000 and the annual average incidence of the cities included in Brazil was around 70 cases per 100,000, a 3% prevalence of PTB is expected in this study. To determine this prevalence with 1% precision, we estimated a sample size of 1,118 individuals, using a 95% confidence interval (Binomial Exact Confidence Interval). Based on these parameters, we expect to include between 37 and 185 cases of PTB among children with LRTI.

A specific sample size was calculated to address the aim related to accuracy, which required a “boost” in sampling by recruiting outpatient participants treated at reference TB centers, considering 20% of PTB prevalence in children/adolescents referred to investigate TB [30]. Similar studies comparing the accuracy of microbiological methods (Ultra and liquid culture) for MTB diagnosis in children and adults reported sensitivities of 75% and 88%, respectively, using Ultra [5,31]. Thus, assuming a sensitivity of 85% and a precision of 0.05, a sample of 226 participants with PTB, confirmed or presumptive, is necessary. Considering a minimum of 37 hospitalized participants, an additional 189 participants are needed. To detect 189 participants who underwent induced sputum collection (nebulization with hypertonic saline solution) with a diagnosis of PTB at outpatient reference TB centers, it is estimated that the screening of 548 participants with signs, symptoms, or radiological exams will meet the inclusion criteria. Considering a loss rate of up to 20% among outpatients, the number of screened participants in this scenario should be 730. It is important to highlight that if the number of hospitalized participants with PTB exceeds 37, fewer outpatients are required if the total number of participants with PTB reaches 226. In summary, the total number of subjects expected for the study (hospitalized and outpatient), considering induced sputum collection, is 1,848 individuals (1,118 hospitalized with LRTI + 730 outpatients).

### Statistical analysis plan

Data normality is assessed using the Shapiro-Wilk normality test. Descriptive analysis is used to characterize the study population, with categorical variables presented as absolute (n) and relative (%) frequencies, normally distributed variables as the mean and standard deviation, and asymmetrically distributed variables as the median and interquartile range.

The diagnostic accuracy will be calculated using the epiR package. The sensitivity, specificity, positive predictive value (PPV), negative predictive value (NPV), and 95% confidence intervals for all estimates will be calculated. Univariate and multivariate logistic modeling are conducted to evaluate the relationship between the outcome (PTB and TB infection) and predictor characteristics (risk factors), as described in Table 1. Odds ratios (ORs) with 95% confidence intervals will be calculated.

Table 2 presents, in a more direct way, the population involved, the statistical analysis plans, and how we will handle missing data for each study aims and outcomes. Statistical analyses are performed in R 4.1.1 (R Foundation for Statistical Computing, Vienna, Austria; http://www.R-project.org), with a significance level of 5% for alpha error (P < 0.05).

### Data sharing

The study protocol, case report forms, consent forms, and assent forms are available in the ReBEC repository, where the study is registered (U1111-1332–7450). The participant-level dataset and statistical codes can be shared upon request after the final analysis is published. In cases of data sharing, the confidentiality of each participant will be ensured by removing data that could identify them. In the case of data sharing, the confidentiality of each participant must be ensured by removing data that could identify them, such as their date and place of birth. Furthermore, this must comply with Brazilian resolutions on the conduct of research, including adherence to the rules for the development of research involving human beings and for data sharing.

### Plan of study monitoring

The data quality collected and reported is systematically monitored for each participating center. Monitoring activities are carried out to verify adherence to Good Clinical Practices and the approved protocol. Remote monitoring focuses on data entry in REDCap and on reviewing regulatory documents. Centralized monitoring identifies missing data and distinguishes between reliable and potentially dubious data. Remote monitoring focuses on data entry in REDCap to identify systematic or significant errors in data collection and reporting, and on reviewing regulatory documents. On-site visits include verification of participant enrollment workflows, document storage and organization, consent and assent data, data consistency between CRFs and REDCap, adverse events, sample storage temperatures, and expiration of supplies. A report with corrective actions is provided following each visit.

### Ethical approval and consent

Ethical approval was obtained by the ethical committee of the proposing institution (Hospital Moinhos de Vento, number 24730819.7.1001.5330). The study at the sites was initiated only after obtaining ethical approval from the local ethics committee. This study adheres to the Regulatory Guidelines and Norms for Research Involving Human Beings, as established by Resolution 466/12 of the Brazilian National Health Council and Law 14874/24, which regulates research involving human beings. Trained researchers obtain informed consent from participants’ legal caregivers before any study procedures. Assent is obtained from children aged 6 years or older who have the capacity to sign it. Study’s consent and assents form are available in ReBEC storage (https://ensaiosclinicos.gov.br/welcome - U1111-1332–7450).

### Risk/harms

The risks include those inherent to the collection of biological material, such as nebulization (induced sputum), secretion aspiration, nasopharyngeal and nasal swabs, and venipuncture. The risks of nebulization for induced sputum include coughing, nasal itching, sneezing, nasal secretion, wheezing, discomfort during nebulization, or malaise. The collection of respiratory secretions (aspiration) can cause local pain, nosebleeds, vomiting, or coughing. The possible reactions are monitored and include: mild epistaxis, increased coughing, and wheezing responsive to an inhaled bronchodilator.

The collection of nasopharyngeal and nasal swabs can cause momentary discomfort, such as nausea, coughing, and tearing (due to the procedure’s cough reflex). Blood collection presents risks, including discomfort, pain, local bleeding, or bruising at the puncture site.

TST application can cause pain and burning at the time of application, and afterward, it can cause itching, local discomfort, local lesions, and blisters (known reactions of the test). These reactions are limited, resolve in a few days, and pose no serious risk to the participant.

To safeguard participant confidentiality and minimize potential risk or harm, participants’ identities remain anonymous, and any personal identifiers are replaced through pseudonymization procedures. Linkage between research data and individual participants will be possible exclusively to authorized members of the research team, who are also involved in the participant’s clinical care. No identifying information will be disclosed in publications or shared datasets.

If an adverse event occurs during study procedures, researchers follow local emergency protocols to ensure participants receive immediate medical attention. The principal investigators at both the local site and the coordinating center receive prompt notification and implement the necessary corrective measures. The study does not provide financial or non-financial incentives to participants.

### Dissemination plan

All results from this study will be published in scientific journals, presented at relevant conferences in the field, and communicated to the study sponsor (the Brazilian Ministry of Health) through a report. Once published, the results will also be widely disseminated in the media.

### Artificial intelligence tools and technologies

Microsoft Copilot and Grammarly were used to support the manuscript’s English revision. The authors revised the tool’s outputs for validity before they were accepted for inclusion in the manuscript. No article contents, data, or supporting files were affected or generated by the use of an artificial intelligence tool.

## Discussion

This study represents the first nationwide investigation into the prevalence of PTB in children and adolescents hospitalized with LRTI in a large country with a high tuberculosis burden, such as Brazil. Additionally, the current study presents an opportunity to collect data in the years after the COVID-19 pandemic, when higher rates of intra-domiciliary transmission are expected. Notably, although Brazil has a recognized high TB burden, it has a significantly lower incidence compared to many countries in Africa and Asia [32]. Previous studies have been retrospective, relying on data from notification systems [15,16]. The present study will provide prospective data from a population with LRTI across multiple centers in Brazil, which may better reflect the true prevalence of PTB in Brazilian children and young adolescents. By addressing this critical gap in the literature, the study may contribute to a greater understanding of PTB epidemiology in pediatric populations with LRTI, providing essential data to guide public health strategies in similar settings.

Beyond the geographical coverage, one of the study’s strengths is its pioneering approach in Brazil, which combines induced sputum collection or tongue swabs and molecular biology techniques, such as Ultra, for diagnosing PTB in children and young adolescents. A significant increase in diagnostic yield has been demonstrated in settings with very high TB incidence in South Africa, reaching a sensitivity higher than 70% [8]. However, as mentioned above, the accuracy of these tests in a setting with intermediate TB incidence may differ significantly. Moreover, this study will provide novel data on the prevalence of TBI in Brazil and the accuracy of pediatric PTB prediction scores used in the country. It will also evaluate the role of IGRAs within these algorithms, which traditionally is based on the TST [20]. Other important data generated by the study will be the prevalence of viral and bacterial pathogens in children and adolescents hospitalized with LRTI, a leading cause of death in children, especially in children under five. Despite the significant progress made during the last decades, LRTIs are still responsible for over 500,000 deaths in under-fives, and close to 50,000 for those from 5 to 14 [33]. This information can serve as a basis for public policies related to immunization and epidemic control of viral and bacterial pathogens among children and adolescents in Brazil.

This study has some potential limitations. First, an inherent challenge remains in the laboratory confirmation of viral and bacterial LRTI, especially in children. Moreover, large multicenter studies investigating the etiology of childhood pneumonia that included asymptomatic controls, such as the PERCH study, have demonstrated that specific pathogens, such as Rhinovirus, are detected at similar rates in both pneumonia cases and controls, making etiological attribution challenging [34]. Second, given that the study’s primary objective in the hospital-based arm is to investigate the prevalence of PTB among patients with LRTI, the coordinating team emphasizes training and monitoring activities to ensure that participants with highly suggestive TB cases are not exclusively recruited, thereby preventing an overestimation of prevalence. Third, the second IS sample from hospitalized participants is not sedimented, which may reduce sensitivity. However, a sensitivity analysis will be conducted, excluding the non-sedimented samples, for the prevalence analysis. Finally, missing data is unavoidable due to the large volume of data and samples collected, as well as the study’s heterogeneous settings and extensive geographical coverage. However, training sessions and monitoring activities are conducted throughout the recruitment period to mitigate these risks. We also accounted for a 20% loss to ensure a sufficient sample size for the study’s primary aim in the outpatient arm.

In conclusion, the results of the TBPed Brazil study will provide critical epidemiological data on PTB and other respiratory pathogens in Brazil, serving as a baseline for future studies and guiding public health policies. Furthermore, based on the findings, the study could serve as a starting point for using a combination of induced sputum collection and molecular biology techniques in public health in Brazil, thereby enhancing the yield of laboratory tests to confirm PTB diagnosis in children and adolescents.

## Supporting information

S1 Table Checklist SPIROS – Standardized protocol items: Recommendations for observational studies. (PDF)

## References

[1] Basile FW, Nabeta P, Ruhwald M, Song R. Pediatric Tuberculosis Diagnostics: Present and Future. J Pediatric Infect Dis Soc. 2022;11(Supplement_3):S85–93. doi: 10.1093/jpids/piac082 36314546 PMC9620430

[2] Gunasekera KS, Marcy O, Muñoz J, Lopez-Varela E, Sekadde MP, Franke MF, et al. Development of treatment-decision algorithms for children evaluated for pulmonary tuberculosis: an individual participant data meta-analysis. Lancet Child Adolesc Health. 2023;7(5):336–46. doi: 10.1016/S2352-4642(23)00004-4 36924781 PMC10127218

[3] Carvalho RF, Carvalho ACC, Velarde LGC, Rossoni AMO, Aurilio RB, Sias SMA, et al. Diagnosis of pulmonary tuberculosis in children and adolescents: comparison of two versions of the Brazilian Ministry of Health scoring system. Rev Inst Med Trop Sao Paulo. 2020;62:e81. doi: 10.1590/S1678-9946202062081 33146310 PMC7608063

[4] Detjen AK, DiNardo AR, Leyden J, Steingart KR, Menzies D, Schiller I, et al. Xpert MTB/RIF assay for the diagnosis of pulmonary tuberculosis in children: a systematic review and meta-analysis. Lancet Respir Med. 2015;3(6):451–61. doi: 10.1016/S2213-2600(15)00095-8 25812968 PMC4756280

[5] Dorman SE, Schumacher SG, Alland D, Nabeta P, Armstrong DT, King B, et al. Xpert MTB/RIF Ultra for detection of Mycobacterium tuberculosis and rifampicin resistance: a prospective multicentre diagnostic accuracy study. Lancet Infect Dis. 2018;18(1):76–84. doi: 10.1016/S1473-3099(17)30691-6 29198911 PMC6168783

[6] Carratalà-Castro L, Munguambe S, Saavedra-Cervera B, de Haas P, Kay A, Marcy O, et al. Performance of stool-based molecular tests and processing methods for paediatric tuberculosis diagnosis: a systematic review and meta-analysis. Lancet Microbe. 2025;6(6):100963. doi: 10.1016/j.lanmic.2024.100963 39547244 PMC12062341

[7] Olbrich L, Franckling-Smith Z, Larsson L, Sabi I, Ntinginya NE, Khosa C, et al. Sequential and parallel testing for microbiological confirmation of tuberculosis disease in children in five low-income and middle-income countries: a secondary analysis of the RaPaed-TB study. Lancet Infect Dis. 2025;25(2):188–97. doi: 10.1016/S1473-3099(24)00494-8 39312914

[8] Zar HJ, Workman LJ, Prins M, Bateman LJ, Mbhele SP, Whitman CB, et al. Tuberculosis Diagnosis in Children Using Xpert Ultra on Different Respiratory Specimens. Am J Respir Crit Care Med. 2019;200(12):1531–8. doi: 10.1164/rccm.201904-0772OC 31381861 PMC6909828

[9] Murray KO, Castillo-Carandang NT, Mandalakas AM, Cruz AT, Leining LM, Gatchalian SR, et al. Prevalence of Tuberculosis in Children After Natural Disasters, Bohol, Philippines. Emerg Infect Dis. 2019;25(10):1884–92. doi: 10.3201/eid2510.190619 31538561 PMC6759243

[10] Yang C, Yasseen AS 3rd, Stimec J, Rea E, Waters V, Lam R, et al. Prevalence of tuberculosis infection and disease in children referred for tuberculosis medical surveillance in Ontario: a single-cohort study. CMAJ Open. 2018;6(3):E365–71. doi: 10.9778/cmajo.20180043 30154220 PMC6182122

[11] Harichander S, Wiafe E, Mensah KB, Bangalee V, Oosthuizen F. The incidence of TB and MDR-TB in pediatrics and therapeutic options: a systematic review. Syst Rev. 2022;11(1):157. doi: 10.1186/s13643-022-02023-1 35927752 PMC9354367

[12] Wang D-M, Li Q-F, Zhu M, Wu G-H, Li X, Xu Y-H, et al. Epidemiological, clinical characteristics and drug resistance situation of culture-confirmed children TBM in southwest of China: a 6-year retrospective study. BMC Infect Dis. 2020;20(1):318. doi: 10.1186/s12879-020-05041-3 32357835 PMC7195785

[13] Villalva-Serra K, Barreto-Duarte B, Rodrigues MM, Queiroz ATL, Martinez L, Croda J, et al. Impact of strategic public health interventions to reduce tuberculosis incidence in Brazil: a Bayesian structural time-series scenario analysis. Lancet Reg Health Am. 2024;41:100963. doi: 10.1016/j.lana.2024.100963 39759249 PMC11697790

[14] BRASIL M da S. Boletim Epidemiológico - Tuberculose 2025. https://www.gov.br/aids/pt-br/central-de-conteudo/boletins-epidemiologicos/2025/boletim-epidemiologico-tuberculose-2025/view

[15] de Oliveira MCB, Sant’Anna CC, Raggio RL, Kritski AL. Tuberculosis among children and adolescents in Rio de Janeiro, Brazil - Focus on extrapulmonary disease. Int J Infect Dis. 2021;105:105–12. doi: 10.1016/j.ijid.2021.02.023 33596481

[16] da Soledade MP, Yamauti SM, Aguiar AS, Sucupira C, Crozatti MTL. Tuberculosis in childhood and adolescence: prevalence and factors associated with treatment abandonment. Cad Saude Publica. 2024;40(9):e00158323. doi: 10.1590/0102-311XPT158323 39292064 PMC11405015

[17] Hogan AB, Jewell BL, Sherrard-Smith E, Vesga JF, Watson OJ, Whittaker C, et al. Potential impact of the COVID-19 pandemic on HIV, tuberculosis, and malaria in low-income and middle-income countries: a modelling study. Lancet Glob Health. 2020;8(9):e1132–41. doi: 10.1016/S2214-109X(20)30288-6 32673577 PMC7357988

[18] Saunders MJ, Evans CA. COVID-19, tuberculosis and poverty: preventing a perfect storm. Eur Respir J. 2020;56(1):2001348. doi: 10.1183/13993003.01348-2020 32444399 PMC7243392

[19] Saunders MJ, Wingfield T, Datta S, Montoya R, Ramos E, Baldwin MR, et al. A household-level score to predict the risk of tuberculosis among contacts of patients with tuberculosis: a derivation and external validation prospective cohort study. Lancet Infect Dis. 2020;20(1):110–22. doi: 10.1016/S1473-3099(19)30423-2 31678031 PMC6928575

[20] Sant’Anna CC, Santos MARC, Franco R. Diagnosis of pulmonary tuberculosis by score system in children and adolescents: a trial in a reference center in Bahia, Brazil. Braz J Infect Dis. 2004;8(4):305–10. doi: 10.1590/s1413-86702004000400006 15565261

[21] Cortez AO, de Melo AC, Neves LO, Resende KA, Camargos P. Tuberculosis in Brazil: one country, multiple realities. J Bras Pneumol. 2021;47(2):e20200119. doi: 10.36416/1806-3756/e20200119 33656156 PMC8332839

[22] World Health Organization. Revised WHO Classification and Treatment of Pneumonia in Children at Health Facilities: Evidence Summaries. Geneva: World Health Organization. 2014. http://www.ncbi.nlm.nih.gov/books/NBK264162/25535631

[23] Graham SM, Ahmed T, Amanullah F, Browning R, Cardenas V, Casenghi M, et al. Evaluation of tuberculosis diagnostics in children: 1. Proposed clinical case definitions for classification of intrathoracic tuberculosis disease. Consensus from an expert panel. J Infect Dis. 2012;205 Suppl 2(Suppl 2):S199-208. doi: 10.1093/infdis/jis008 22448023 PMC3334506

[24] World Health Organization. WHO operational handbook on tuberculosis: module 5: management of tuberculosis in children and adolescents. 2023. https://www.who.int/publications/i/item/9789240046832 35404556

[25] Fancourt N, Deloria Knoll M, Barger-Kamate B, de Campo J, de Campo M, Diallo M, et al. Standardized Interpretation of Chest Radiographs in Cases of Pediatric Pneumonia From the PERCH Study. Clin Infect Dis. 2017;64(suppl_3):S253–61. doi: 10.1093/cid/cix082 28575359 PMC5447844

[26] Brasil, Ministério da Saúde. Nota informativa No 7/2022-CGDR/.DCCI/SVS/MS. 2022. https://www.saude.mg.gov.br/wp-content/uploads/2025/01/sei_ms_-_0028087336_-_nota_informativa.pdf

[27] Harris PA, Taylor R, Minor BL, Elliott V, Fernandez M, O’Neal L, et al. The REDCap consortium: Building an international community of software platform partners. J Biomed Inform. 2019;95:103208. doi: 10.1016/j.jbi.2019.103208 31078660 PMC7254481

[28] Oliwa JN, Karumbi JM, Marais BJ, Madhi SA, Graham SM. Tuberculosis as a cause or comorbidity of childhood pneumonia in tuberculosis-endemic areas: a systematic review. The Lancet Respiratory Medicine. 2015;3(3):235–43. doi: 10.1016/s2213-2600(15)00028-4 25648115

[29] Moore DP, Klugman KP, Madhi SA. Role of Streptococcus pneumoniae in hospitalization for acute community-acquired pneumonia associated with culture-confirmed Mycobacterium tuberculosis in children: a pneumococcal conjugate vaccine probe study. Pediatr Infect Dis J. 2010;29(12):1099–04. doi: 10.1097/inf.0b013e3181eaefff 21155174

[30] Maciel ELN, Dietze R, Silva RECF, Hadad DJ, Struchiner CJ. Evaluation of a scoring system recommended by the Brazilian Ministry of Health for the diagnosis of childhood tuberculosis. Cad Saude Publica. 2008;24(2):402–8. doi: 10.1590/s0102-311x2008000200019 18278287

[31] Nicol MP, Workman L, Prins M, Bateman L, Ghebrekristos Y, Mbhele S, et al. Accuracy of Xpert Mtb/Rif Ultra for the Diagnosis of Pulmonary Tuberculosis in Children. Pediatr Infect Dis J. 2018;37(10):e261–3. doi: 10.1097/INF.0000000000001960 29474257

[32] World Health Organization. Global Tuberculosis Report 2024. https://www.who.int/teams/global-tuberculosis-programme/tb-reports/global-tuberculosis-report-2024

[33] GBD 2021 Lower Respiratory Infections and Antimicrobial Resistance Collaborators. Global, regional, and national incidence and mortality burden of non-COVID-19 lower respiratory infections and aetiologies, 1990-2021: a systematic analysis from the Global Burden of Disease Study 2021. Lancet Infect Dis. 2024;24(9):974–1002. doi: 10.1016/S1473-3099(24)00176-2 38636536 PMC11339187

[34] Pneumonia Etiology Research for Child Health (PERCH) Study Group. Causes of severe pneumonia requiring hospital admission in children without HIV infection from Africa and Asia: the PERCH multi-country case-control study. Lancet. 2019;394(10200):757–79. doi: 10.1016/S0140-6736(19)30721-4 31257127 PMC6727070

