# Supplementary material for: Nationwide epidemiological study of tuberculosis and other respiratory pathogens among children and adolescents in Brazil: TBPed Brazil study protocol
Source: PLoS One. 2026 Feb 12;21(2):e0342753. doi: 10.1371/journal.pone.0342753 (PMC12900370; doi:10.1371/journal.pone.0342753)
Supplement: S1 Table — (PDF) [file pone.0342753.s001.pdf]

S1 Table. Checklist SPIROS – Standardized Protocol Items: Recommendations for Observational Studies.

☑ SPIROS

Standardized Protocol Items Recommendations for Observational Studies

**Section/Item**	**Item Number**	**Description**	**Page**
**Part A: General information**			
Title	1	Descriptive title Identifying study design in the title	1
Protocol version	2	Version or amendment number with date and summary of the changes	NA
Protocol summary	3	An informative and balanced summary of the study protocol	2 to 3
Sponsor and funder details	4	Name of Sponsor and funder and types of financial material and other support	Reported in Funding Statement form
Conflict of interest statements	5	Statement about any financial and other competing interests for principal or co-investigators for the overall study	Reported in submission form
Investigators name	6a	Names of the principal and co-investigators	1
Affiliation of investigators	6b	Affiliated institutions of the investigators	1 to 2
Principal researcher/s contact detail	6c	Name e-mail address affiliation of principal researcher	2
**Part B: Introduction**			
Background of the study	7a	Description of research question and scientific background of the study	3 to 4
Review of prior research	7b	Summary of relevant existing research (published or unpublished)	4
Rationale of study	7c	Justification for conducting the study	4
Aim	8a	Broader aims and overall objective	5
Objectives of the study	8b	Primary and secondary objectives including any prespecified hypothesis	5
Objectives of the study	8c	Specify whether the intention is to (a) estimate causal effects (b) predict outcomes or (c) simple description	5
**Part C: Methods**			
Study design	9a	Description of study design (case control cross-sectional or cohort) and type of study	5
Study setting	9b	Description of the study setting and detail of precise locations of the study sites	5
Study schedule	10a	Description of the expected schedule of the study including relevant dates and periods	5

Study schedule	10b	Figure or table describing expected time frame for each step	NA
Sample size	11	Estimation of minimum sample size required with justifications	19 to 20
Sampling procedure	12	Detailed description of the sampling frame and sampling strategy	19 to 20
**Participant selection**			
Participant selection (cohort)	13a	Inclusion/exclusion criteria and methods of participant selection	NA
Participant selection (case-control)	13b	Inclusion/exclusion criteria case ascertainment and control selection	NA
Participant selection (cross-sectional)	13c	Inclusion/exclusion criteria and methods of participant selection	7
Variables	14a	Detailed description of baseline and outcome variables exposures, predictors, confounders	8 to 12, and tables 1 and 2
Data sources/measurement	14b	Sources of data and details of assessment/measurement methods	8 to 12, and tables 1 and 2
Data collection and management	15a	Plans for assessment and collection of outcomes and study data	13 to 18
	15b	Description of data collection methods	13 to 18, and figure 1
	15c	Processes to promote data quality during data collection	7, 13 and 21
	15d	Description of study instruments and their reliability/validity	8 to 18
	15e	Plans for data entry coding security and storage	18
	15f	Reference to where details of data management procedures can be found	18
Blinding procedure	16	Description of blinding procedure (if applicable)	18
Potential bias	17	Description of potential biases and plan to minimize them	24 to 25, and table 2
Statistical analysis plan	18	Detailed description of methods for analysing and presenting outcomes	20 and table 2
Handling of missing data	19	Methods to handle missing data (e.g. multiple imputation)	20 and table 2
Withdrawals/lost to follow-up	20a	Procedures when a participant ceases participation or is lost to follow-up	20 and table 2
Replacements	20b	Plans for replacement or substitution of withdrawn participants	NA
Outcome	21	Definition and description of all outcomes	10 to 12 and table 2
Data confidentiality	22	Process to ensure data confidentiality	22
Follow up	23	Plan of follow up including schedule and methods	NA
Study monitoring	24	Plan for study monitoring and independence from investigators/sponsors	21
Training of surveyors	25	Description of training for investigators and surveyors	7

Quality assurance	26	Plan of quality assurance and back-checking data collection	21
**Part D: Ethical consideration**			
Ethical approval	27a	Plan for seeking ethics approval from committees/boards	21-22
	27b	Justification if ethics approval will not be sought	NA
Consent and assent	28a	Who will obtain consent/assent and how	21
	28b	Reason if consent/assent not sought	NA
	28c	Reference to consent forms and translations	22
Risk/harm to participants	29a	Detailed description of potential risks or harms	22
	29b	Plans for collecting assessing reporting adverse events	22
	29c	Statement on anonymity/pseudonymization/linkage to participants	22
	29d	Plans for giving incentives	22
Adverse event reporting	30	Outline how adverse events and serious adverse events will be collected/reported	22
Patient/participant involvement	31	Statement on involvement of patients/participants in protocol development	NA
**Part E. Repor ng and dissemina on**			
Dissemination/publication plan	32a	Plans to communicate study results to boards participants stakeholders public	23
	32b	Methods to communicate findings (publication databases data-sharing)	23 and 20-21
	32c	Authorship eligibility guidelines (e.g. ICMJE recommendations)	Reported in the submission form
**Part F: Others**			
AI-assisted technology use	33	Disclosure of AI-assisted technologies in protocol writing	23
AI-assisted technology use	34	Name of AI tools used and statement on review/editing	23
References	35	Complete list of references cited in protocol	25 to 28
Funding	36	Source of funding and role of funders	Reported in Funding Statement form
Open science (registration)	37a	Study identifier and registry name	20 to 21
	37b	Plans for public access to protocol dataset analysis plan codes, study materials	20 to 21
